# Supplementary material for: Histone H3K36me2 and H3K36me3 form a chromatin platform essential for DNMT3A-dependent DNA methylation in mouse oocytes
Source: Nat Commun. 2022 Aug 3;13:4440. doi: 10.1038/s41467-022-32141-2 (PMC9349174; doi:10.1038/s41467-022-32141-2)
Supplement: Supplementary file 1 — Supplementary information [file 41467_2022_32141_MOESM1_ESM.pdf]

Supplementary Fig. 1

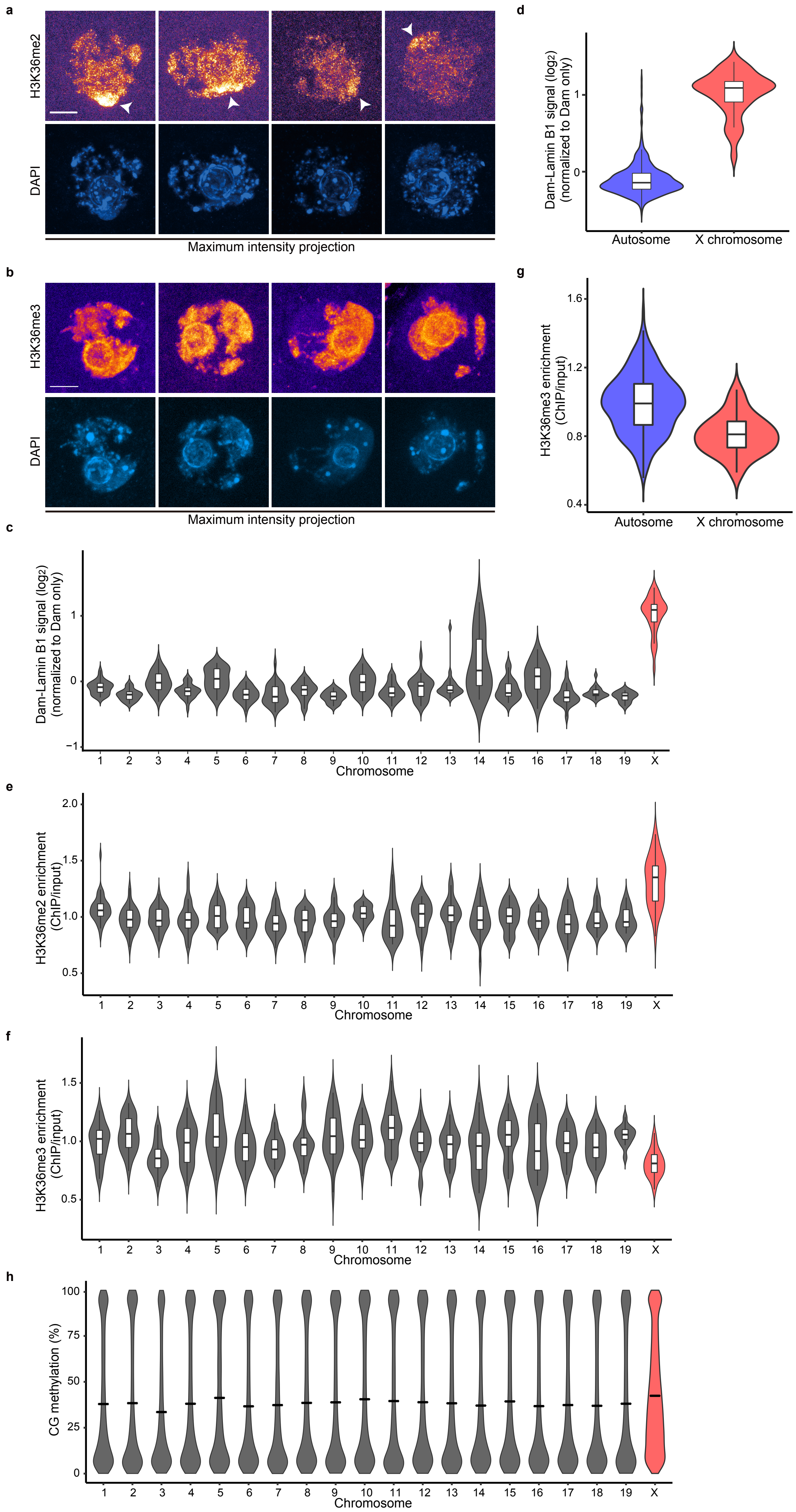

Supplementary Fig. 1: X chromosome possesses globally high H3K36me2 enrichment.

a, Representative images of germinal vesicles of FGOs immunostained for H3K36me2. Images of the four FGOs. Arrowheads indicate strong H3K36me2 signals near the nuclear envelope. The maximum intensity projection images are shown. Scale bar, 10 μm.

b, Representative images of germinal vesicles of FGOs immunostained for H3K36me3. Images of the four FGOs. Scale bar, 10 μm.

c, Violin plots showing Dam-Lamin B1 signal levels in individual chromosomes. The data were normalized to Dam-only data in each 5-Mb bin[19] (n= 40, 37, 33, 32, 31, 30, 30, 26, 25, 27, 25, 25, 25, 25, 21, 20, 19, 19, and 13 bins for chromosome 1, 2, …, and 19, respectively, and n= 35 bins for chromosome X). Boxplots show median value and 25–75th percentiles, and whiskers show 1.5× interquartile range from the box. Source data are provided as a Source Data file.

d, Violin plots showing Dam-Lamin B1 signal levels of 5-Mb bins in autosomes (n = 503) and the X chromosome (n = 35)[19]. Boxplots show median value and 25–75th percentiles, and whiskers show 1.5× interquartile range from the box. Source data are provided as a Source Data file.

e, Violin plots showing H3K36me2 enrichment in 5-Mb bins in individual chromosomes (n= 40, 37, 32, 32, 31, 30, 30, 26, 25, 27, 25, 25, 25, 25, 21, 20, 19, 19, and 13 bins for chromosome 1, 2, …, and 19, respectively, and n= 35 bins for chromosome X). Boxplots show median value and 25–75th percentiles, and whiskers show 1.5× interquartile range from the box. Source data are provided as a Source Data file.

f, Violin plots showing H3K36me3 enrichment in 5-Mb bins in individual chromosomes (n= 40, 37, 32, 32, 31, 30, 30, 26, 25, 27, 25, 25, 25, 25, 21, 20, 19, 19, and 13 bins for chromosome 1, 2, …, and 19, respectively, and n= 35 bins for chromosome X). Boxplots show median value and 25–75th percentiles, and whiskers show 1.5× interquartile range from the box. Source data are provided as a Source Data file.

g, Violin plots showing H3K36me3 enrichment in 5-Mb bins in autosomes (n = 502) and X chromosome (n = 35). Boxplots show median value and 25–75th percentiles, and whiskers show 1.5× interquartile range from the box. Source data are provided as a Source Data file.

h, Violin plots showing CG methylation levels of 10-kb bins in individual chromosomes (n= 19,191, 17,638, 15,612, 15,010, 14,673, 14,592, 13,734, 12,541, 12,115, 12,685, 11,859, 11,646, 11,673, 11,848, 10,067, 9,495, 9,155, 8,737, and 5,813 bins for chromosome 1, 2, …, and 19, respectively, and n= 15,494 bins for chromosome X). Horizontal bars indicate mean values.

Supplementary Fig. 2

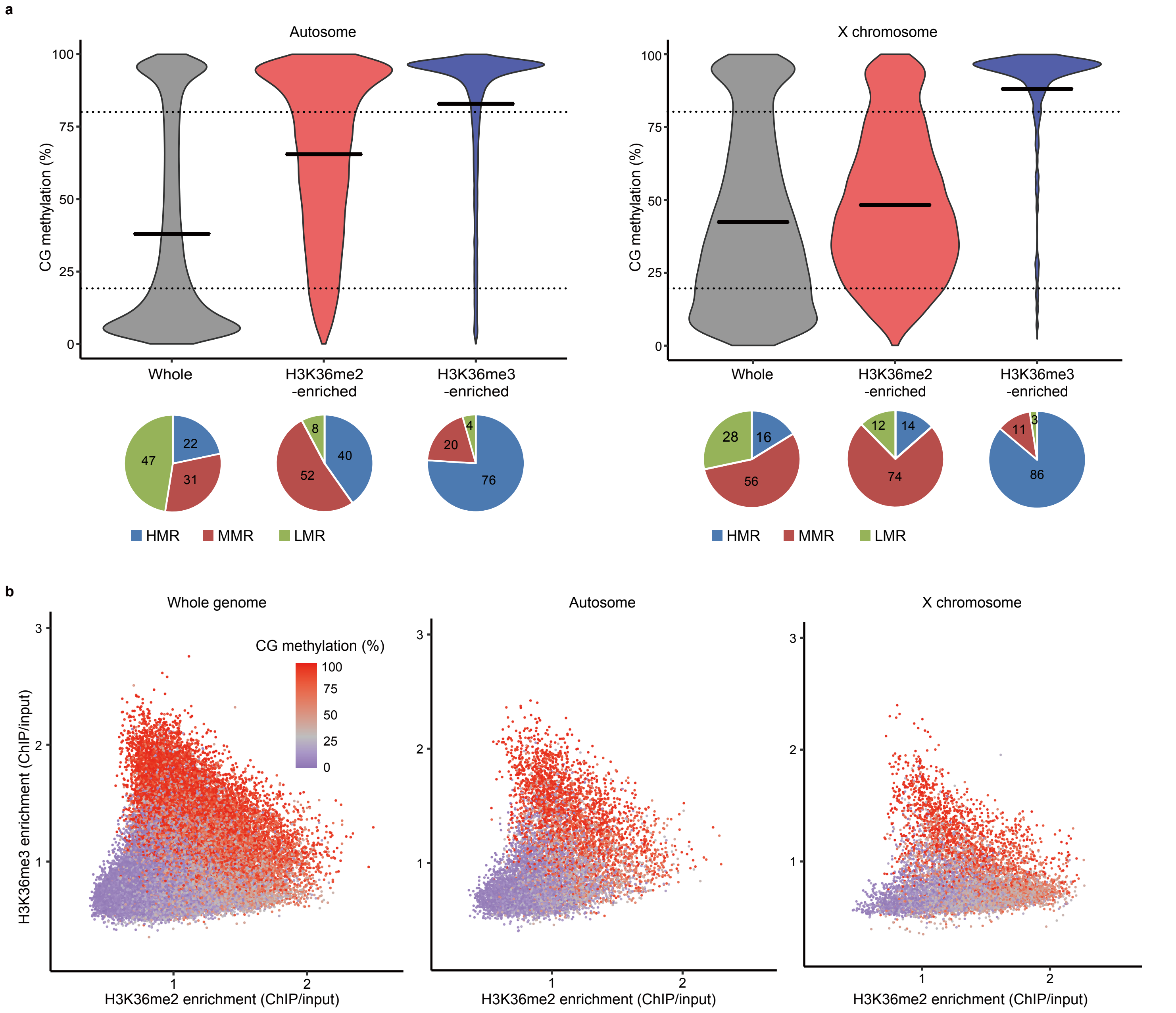

Supplementary Fig. 2: H3K36me2-enriched regions are associated with moderate levels of CG methylation.

a, Violin plots showing CG methylation levels of 10-kb bins from all autosomes (n = 238,084) and their H3K36me2-enriched (n = 23,268) and H3K36me3-enriched regions (n = 28,165) (left). A similar analysis was performed for the X chromosome: 10-kb bins from the entire X chromosome (n = 15,494) and its H3K36me2-enriched (n = 6,966) and H3K36me3-enriched regions (n = 490). Horizontal bars indicate mean values. Pie charts show percentages of 10-kb bins categorized as HMRs, MMRs, and LMRs.

b, Scatter plots showing reciprocal H3K36me2 and H3K36me3 enrichment with a color gradient for CG methylation levels in control FGOs. Fifty thousand randomly selected 10-kb bins were plotted for the whole genome (left) and ten thousand of those for autosomes (middle) and X chromosomes (right).

Supplementary Fig. 3

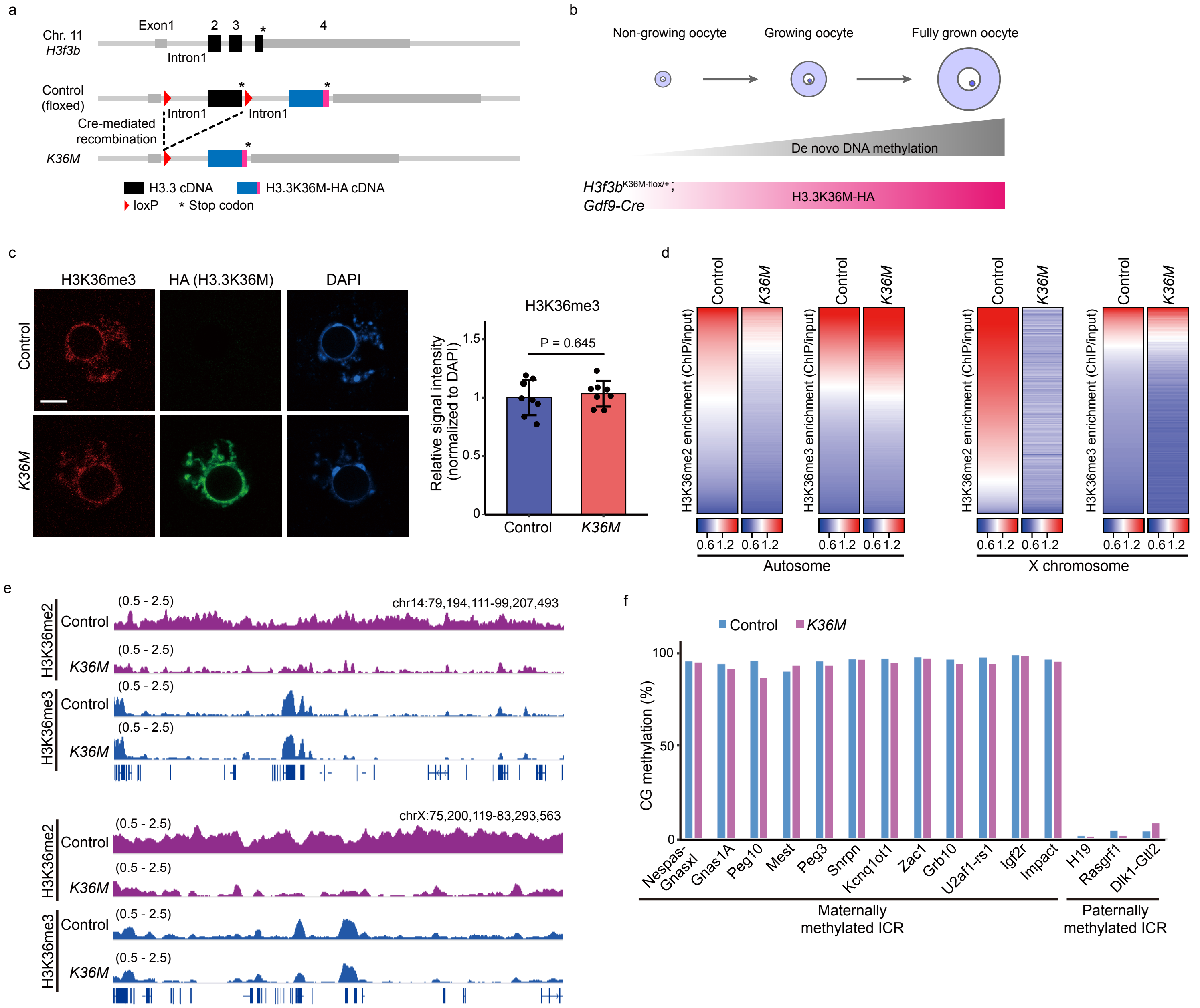

Supplementary Fig. 3: Expression of H3.3K36M causes loss of H3K36me2.

a, Scheme showing Cre-mediated recombination strategy. H3.3K36M-HA indicates H3.3K36M protein tagged with HA peptides at the carboxy terminus.

b, Chronology of oocyte growth, de novo DNA methylation, and H3.3K36M-HA expression driven by *Gdf9*-Cre.

c, Representative images of control and *K36M* FGOs immunostained for H3K36me3 (left) and plots showing their signal intensities (right). H3.3K36M-HA expression was confirmed through HA immunostaining. Signal intensity was measured in the control (n = 8) and *K36M* FGOs (n = 8) from two independent experiments. Scale bar, 10 μm. P-values (two-tailed Mann-Whitney U tests) are indicated. Error bars indicate the mean ± SD. Source data are provided as a Source Data file.

d, Heatmaps showing H3K36me2 and H3K36me3 enrichment in 10-kb bins for the autosomes (left) and X chromosome (right) in control and *K36M* FGOs. Enrichment values were normalized using the spike-in control.

e, Genome browser snapshots showing H3K36me2 and H3K36me3 enrichment in control and *K36M* FGOs. Representative regions from chromosome 14 (left) and X (right). The enrichment values were ChIP/input.

f, CG methylation levels in imprinting control regions (ICRs) in control and *K36M* FGOs. Source data are provided as a Source Data file.

Supplementary Fig. 4

a

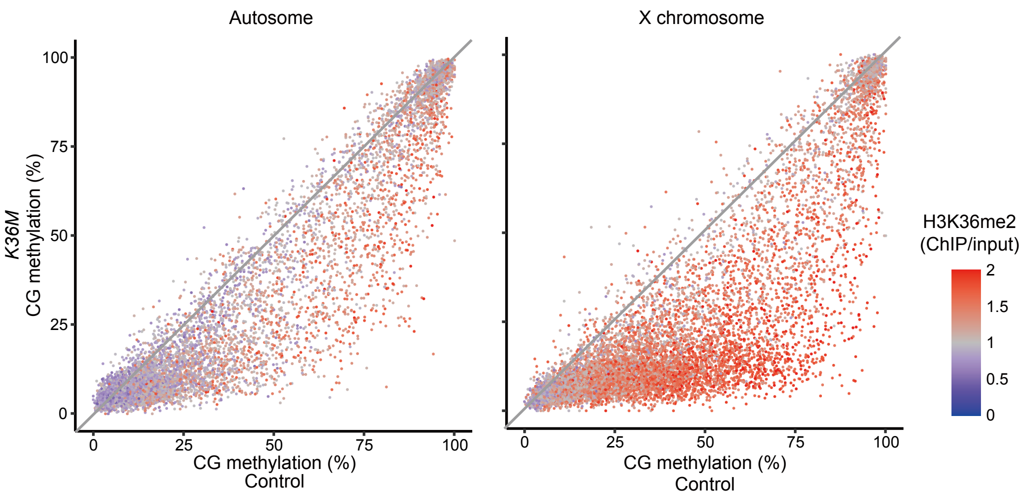

b

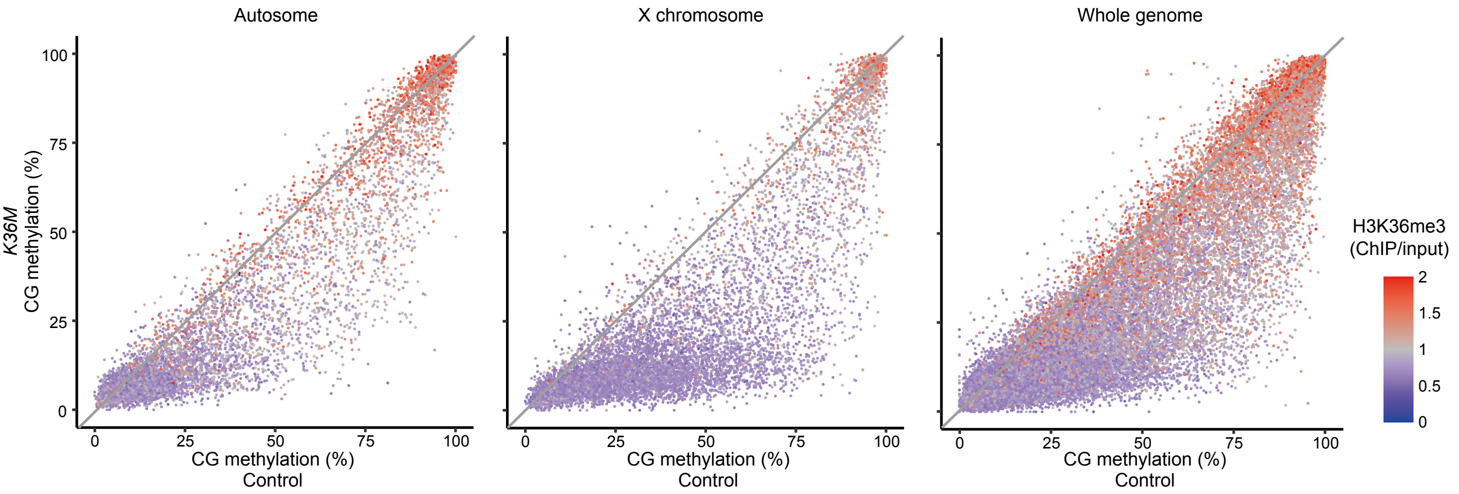

Supplementary Fig. 4: Loss of H3K36me2 causes CG hypomethylation in MMRs.

a, Scatter plots showing CG methylation levels in control and *K36M* FGOs in autosomes (left) and X chromosomes (right). Ten thousand randomly selected 10-kb bins were plotted with a color gradient for H3K36me2 enrichment in control FGOs.

b, Scatter plots showing CG methylation levels in control and *K36M* FGOs across the whole genome (left), autosomes (middle), and X chromosome (right). Fifty thousand (left) and ten thousand (middle and right) randomly selected 10-kb bins were plotted with a color gradient for H3K36me3 enrichment in control FGOs.

Supplementary Fig. 5

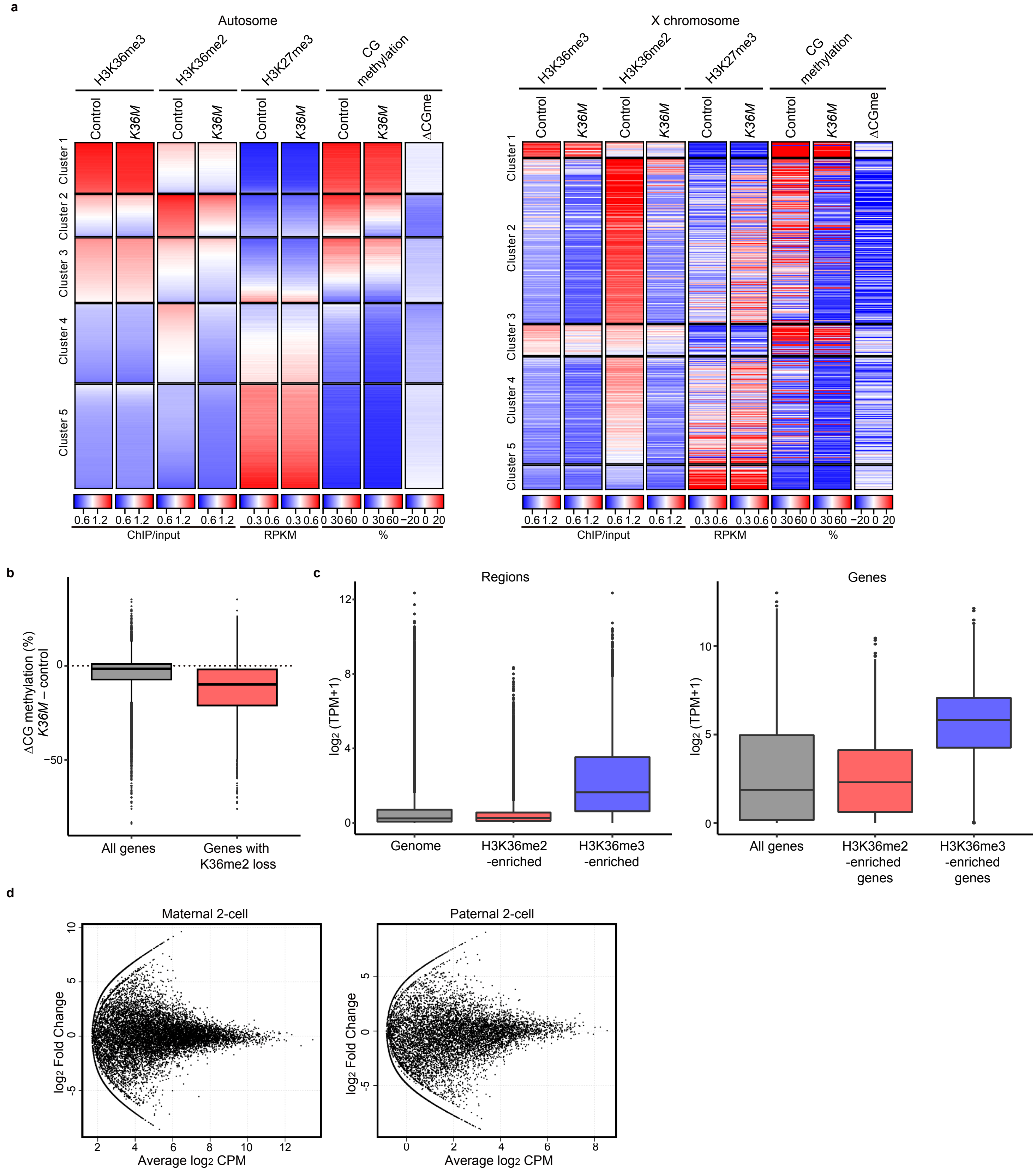

Supplementary Fig. 5: Loss of H3K36me2 affects H3K27me3 and CG methylation especially in X chromosome but has little impact on the transcriptome of 2-cell embryos.

a, Heatmaps showing H3K36me3, H3K36me2, and H3K27me3 enrichment and CG methylation levels in 10-kb bins in control and *K36M* FGOs. CG methylation differences between *K36M* and control FGOs (ΔCGme). The analysis was performed separately for autosomes (left) and X chromosomes (right).

b, Box plots showing CG methylation changes between *K36M* and control FGOs for all genes (left, n = 22,552) and those with H3K36me2 loss (right, n = 2,299). The box shows the median value and 25–75th percentiles, and whiskers show 1.5× interquartile range from the box.

c, Box plots showing the levels of transcripts mapped to 10-kb bins across the genome (n = 253,578), H3K36me2-enriched regions (ChIP/input ≥ 1.4, n = 30,234), and H3K36me3-enriched regions (ChIP/input ≥ 1.5, n = 28,655) in control FGOs (left). Box plots showing expression levels of all genes (n = 22,436), H3K36me2-enriched genes (ChIP/input ≥ 1.2, n = 4,687), and H3K36me3-enriched genes (ChIP/input ≥ 1.4, n = 4,342) in control FGOs (right). The H3K36me2-enriched and H3K36me3-enriched genes are listed in Supplementary Data 2 and 3. The box shows the median value and 25–75th percentiles, and whiskers show 1.5× interquartile range from the box. TPM, transcripts per kilobase million.

d, MA plots showing changes in gene expression between K36M oocyte-derived and control late 2-cell embryos. Transcripts from maternal (left) and paternal (right) alleles were analyzed separately. Genes with FDR < 0.05 were not detected.

Supplementary Fig. 6

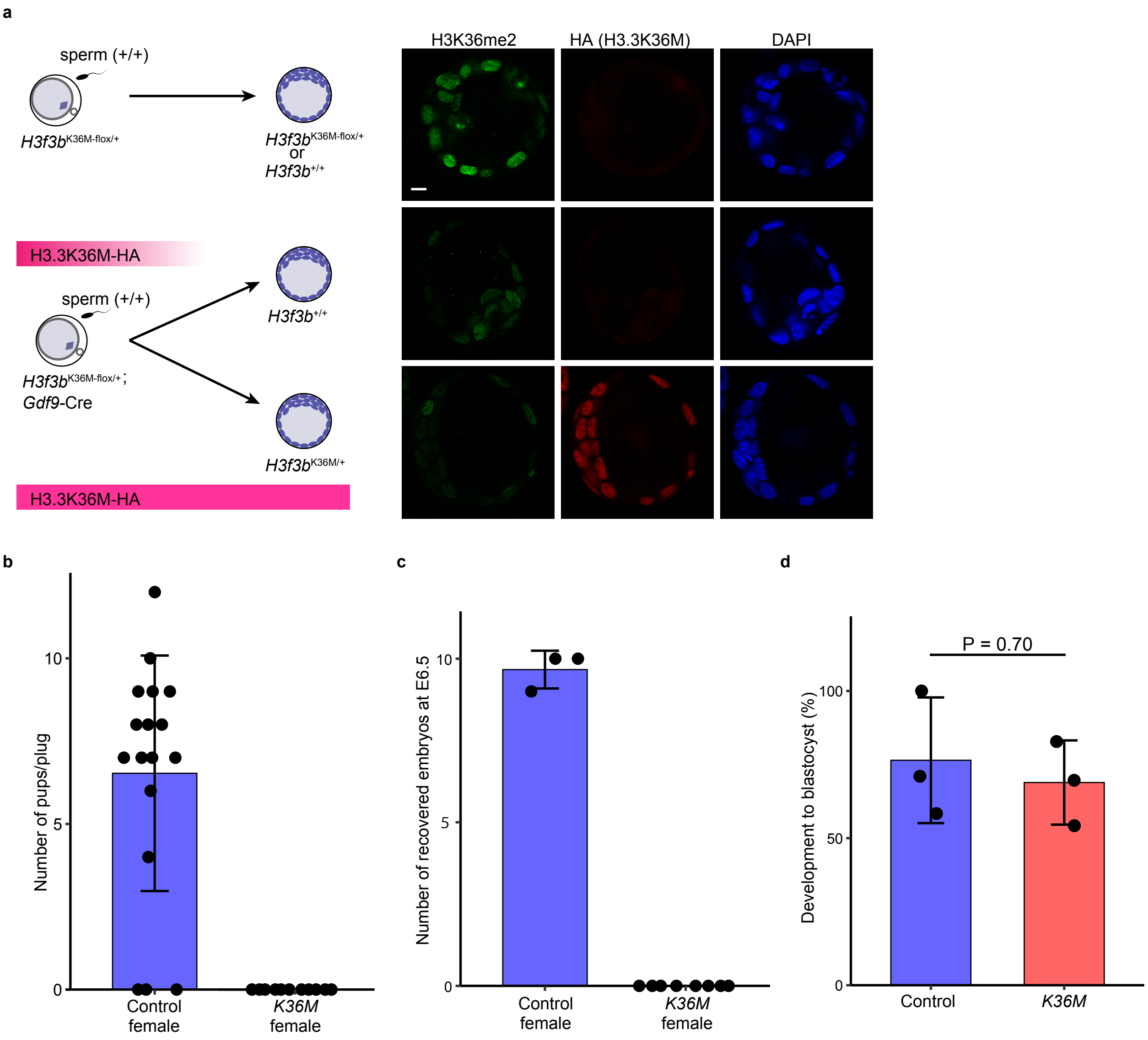

Supplementary Fig. 6: *K36M* oocytes lead to embryonic lethality around implantation.

a, Representative images of blastocysts immunostained for H3K36me2 and HA. A schematic representation of the experimental outline and genotypes is shown on the left. Control (n = 23) and *K36M* (n = 36) oocytes were fertilized with wild-type sperm in two independent experiments. Scale bar, 10 μm.

b, Plots showing the number of pups per plug for control (n = 17) and *K36M* females (n = 11) crossed with wild-type males. Error bars indicate the mean ± SD. Source data are provided as a Source Data file.

c, Plots showing the numbers of embryos recovered at E6.5 from control (n = 3) and *K36M* females (n = 8) after crossing with wild-type males. Error bars indicate the mean ± SD. Source data are provided as a Source Data file.

d, Plots showing blastocyst formation rates Control (n = 84, 69, and 66) and *K36M* oocytes (n = 83, 64, and 56) were fertilized with wild-type sperm in three independent experiments. P-values (two-tailed Mann-Whitney U tests) are shown. Error bars indicate the mean ± SD. Source data are provided as a Source Data file.

Supplementary Fig. 7

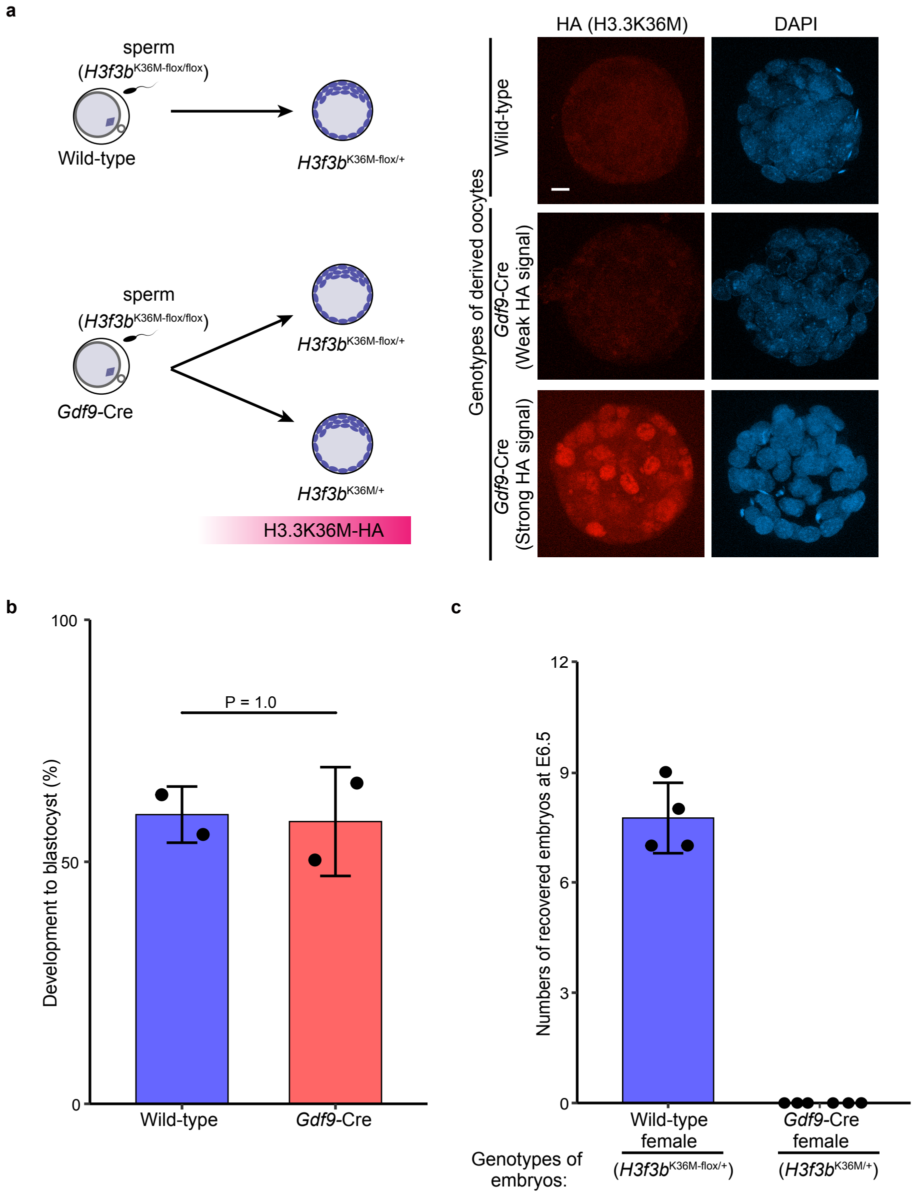

Supplementary Fig. 7: Zygotic H3.3K36M leads to embryonic lethality around implantation.

a, Representative images of blastocysts immunostained for HA. A schematic representation of the experimental outline and genotypes is shown on the left. Wild-type (n = 24) and *Gdf9*-Cre oocytes (n = 28) were fertilized with *H3f3b*$^{K36M\text{-}flox/flox}$ sperm in two independent experiments. Blastocysts derived from *Gdf9*-Cre oocytes showed variations in H3.3K36M expression: signal measurement identified those with weak to moderate HA expression (n = 21) and those with strong HA expression (n = 7). The maximum intensity projection images are shown. Scale bar, 10 µm.

b, Plots showing blastocyst formation rates. Wild-type (n = 166 and 124) and *K36M* oocytes (n = 83 and 135) were fertilized with *H3f3b*$^{K36M\text{-}flox/flox}$ sperm in two independent experiments. P-values (two-tailed Mann-Whitney U tests) are shown. Error bars indicate the mean ± SD. Source data are provided as a Source Data file.

c, Plots showing the numbers of embryos recovered at E6.5, from wild-type (n = 4) and *Gdf9*-Cre females (n = 6) crossed with *H3f3b*$^{K36M\text{-}flox/flox}$ males. The genotypes of derived embryos are also shown (bottom). Error bars indicate the mean ± SD. Source data are provided as a Source Data file.

Supplementary Fig. 8

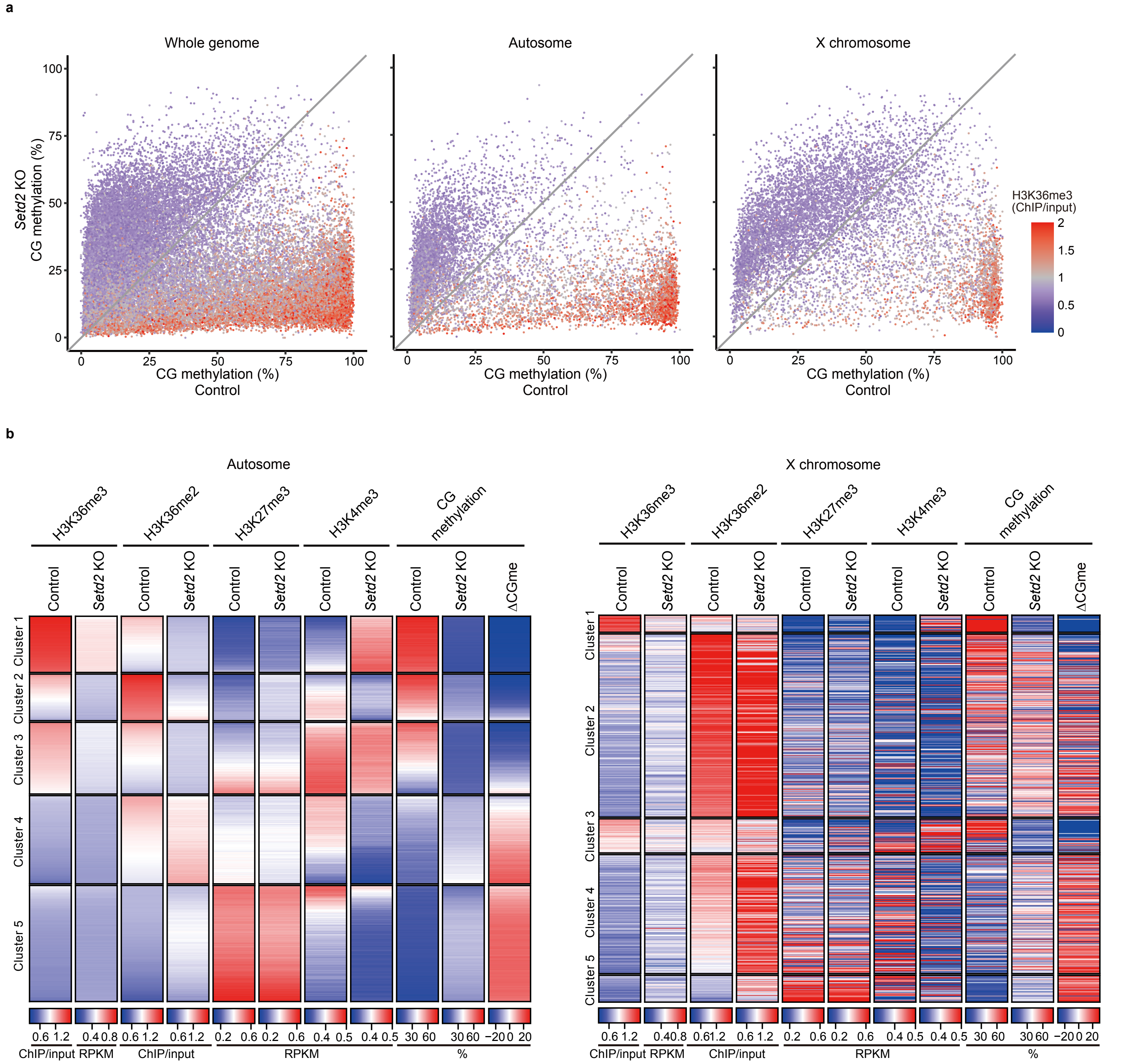

Supplementary Fig. 8: Loss of H3K36me3 results in excessive gain of CG methylation in regions with specific histone marks.

a, Scatter plots showing CG methylation levels in control and *Setd2* KO FGOs across the whole genome (left), autosomes (middle), and X chromosomes (right). Fifty thousand (left) and ten thousand randomly selected 10-kb bins (middle and right) were plotted with a color gradient for H3K36me3 enrichment in control FGOs.

b, Heatmaps showing H3K36me3, H3K36me2, H3K27me3, and H3K4me3 enrichment and CG methylation of 10-kb bins in control and *Setd2* KO FGOs[11, 14]. CG methylation differences between *Setd2* KO and control FGOs (ΔCGme). The analysis was performed separately for autosomes (left) and X chromosomes (right). The data are sorted in the same order as that in Supplementary Fig. 5a.

Supplementary Fig. 9

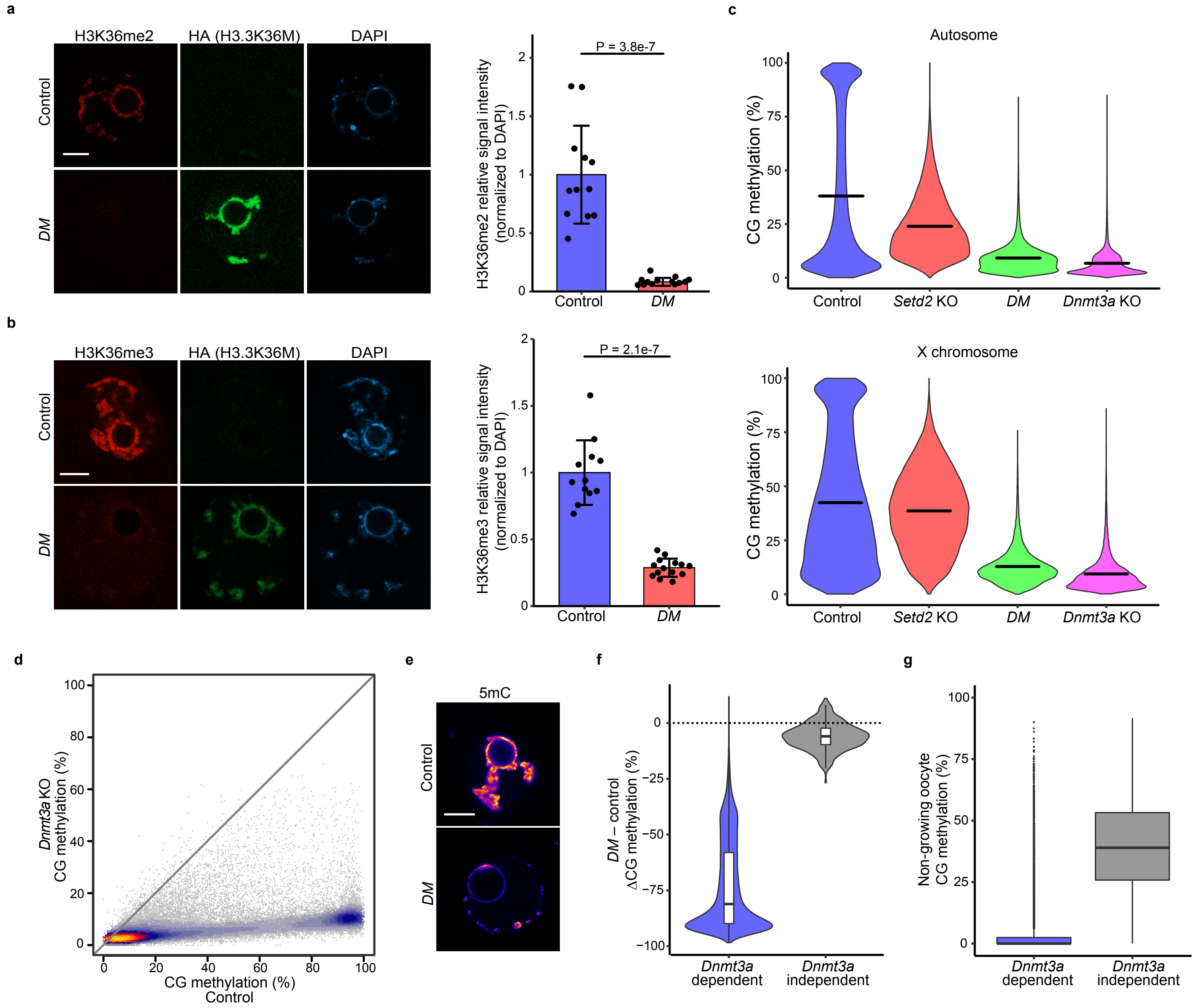

Supplementary Fig. 9: Depletion of both H3K36me2 and H3K36me3 results in global hypomethylation in FGOs.

a, Representative images of control and *DM* FGOs immunostained for H3K36me2 (left) and plots showing signal intensities (right). H3.3K36M-HA expression was confirmed using HA immunostaining. Signal intensity was measured in the control (n = 12) and *DM* FGOs (n = 13) from two independent experiments. Scale bar, 10 μm. P-values (two-tailed Mann-Whitney U tests) are indicated. Error bars indicate the mean ± SD. Source data are provided as a Source Data file.

b, Representative images of control and *DM* FGOs immunostained for H3K36me3 (left) and plots showing signal intensities (right). H3.3K36M-HA expression was confirmed using HA immunostaining. Signal intensity was measured in the control (n = 12) and *DM* FGOs (n = 14) from two independent experiments. Scale bar, 10 μm. P-values (two-tailed Mann-Whitney U tests) are indicated. Error bars indicate the mean ± SD. Source data are provided as a Source Data file.

c, Violin plots showing CG methylation levels in 10-kb bins of autosomes (left) and X chromosome (right) in control, *Setd2* KO, *DM*, and *Dnmt3a* KO[7] FGOs.

Horizontal bars indicate mean values.

d, Scatter plots showing CG methylation levels in the control and *Dnmt3a* KO[7] FGOs. Fifty thousand randomly selected 10-kb bins were plotted.

e, Representative images of control and *DM* FGOs immunostained for 5mC. Control (n = 9) and *DM* FGOs (n = 20) were studied in two independent experiments. Scale bar, 10 μm.

f, Violin plots showing CG methylation differences between *DM* and control FGOs in 10-kb bins of *Dnmt3a*-dependent (n = 59,806) and *Dnmt3a*-independent regions (n = 262)[7]. Boxplots show median value and 25–75th percentiles, and whiskers show 1.5× interquartile range from the box.

g, Box plots showing CG methylation levels in non-growing oocytes[7] in 10-kb bins of *Dnmt3a*-dependent (n = 59,806) and *Dnmt3a*-independent regions (n = 262)[7]. The box shows the median value and 25–75th percentiles, and whiskers show 1.5× interquartile range from the box.

Supplementary Note

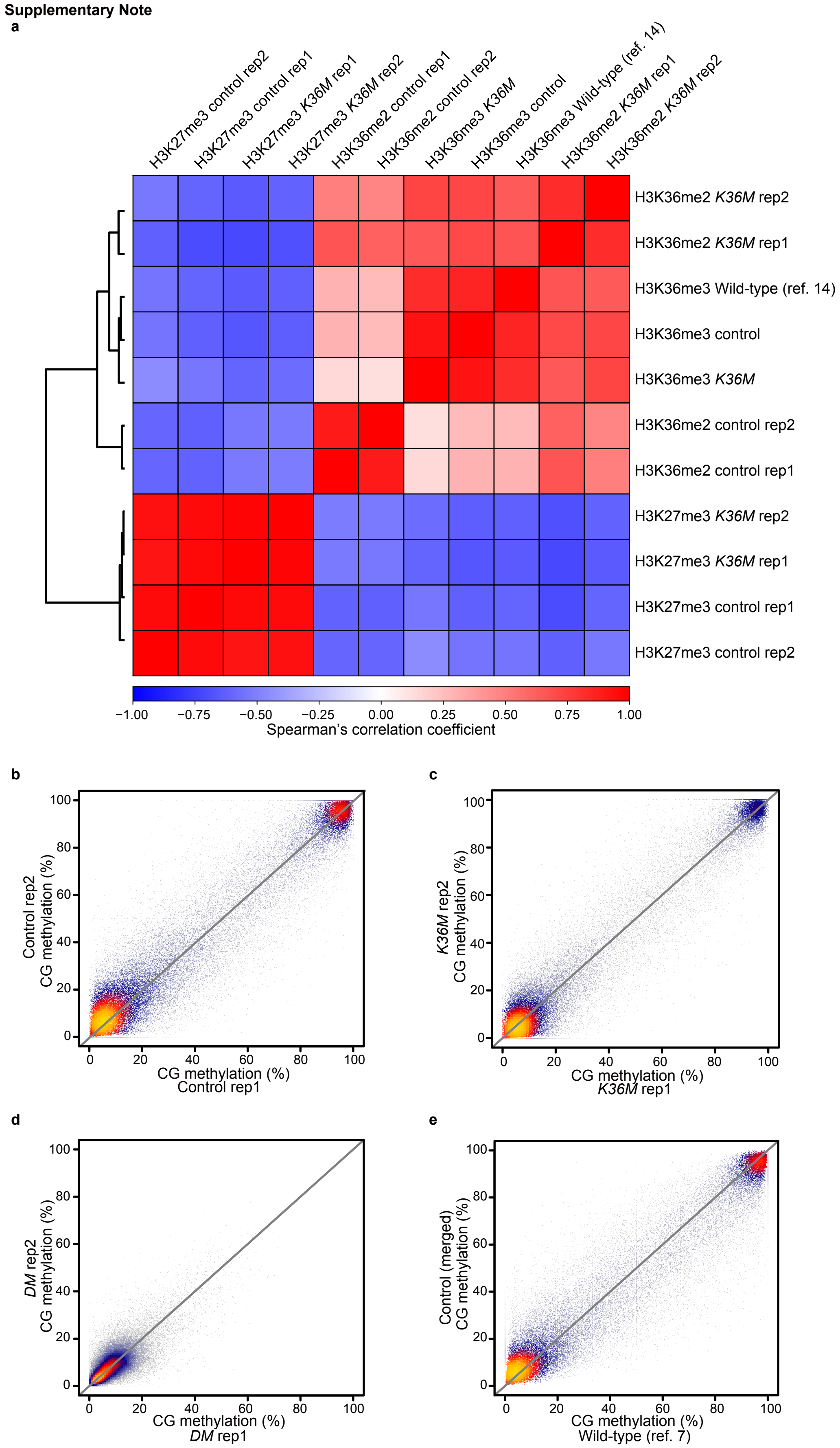

Supplementary Note: Reproducibility between replicates of ChIP-seq, CUT&RUN, and WGBS data.

a, Hierarchical clustering and correlation analysis of histone modification data. Correlation was determined using enrichment in 10-kb bins. Previous H3K36me3 ChIP-seq data[14] were also used to confirm the validity of our data. Heatmaps show Spearman's correlation coefficients between samples.

b, Scatter plots showing CG methylation levels in replicates 1 and 2 of control FGOs. Fifty thousand randomly selected 10-kb bins were plotted.

c, Scatter plots showing CG methylation levels in replicates 1 and 2 of *K36M* FGOs. Fifty thousand randomly selected 10-kb bins were plotted.

d, Scatter plots showing CG methylation levels in replicates 1 and 2 of the *DM* FGOs. Fifty thousand randomly selected 10-kb bins were plotted.

e, Scatter plots showing CG methylation levels in witd-type[7] and control FGOs (replicates 1 and 2 were merged). Fifty thousand randomly selected 10-kb bins were plotted.

Supplementary Table 1. List of publicly available data reanalyzed in this study.

Data	Accession Number	Reference	Related figures
Dam-Lamin B1 signal in FGO	GSE112551	19	Supplementary Fig. 1c,d
H3K36me2 in *Setd2* KO FGO	GSE148150	11	Fig. 4b,e and Supplementary Fig. 8b
H3K4me3, H3K27me3, and H3K36me3 in *Setd2* KO and control FGO	GSE112835	14	Fig. 4b, Supplementary Fig. 8b and Supplementary Note
H3K4me3 and CG methylation in *Mll2* KO and control FGO	GSE93941	12	Fig. 4c,d
CG methylation in non-growing oocyte and *Dnmt3a* KO FGO	DRA000570	7	Fig. 5b,c,d,f,g, Supplementary Fig. 9c,d and Supplementary Note

Supplementary Table 2. Summary of ULI-NChIP-seq, CUT&RUN, and RNA-seq.

Sample	Total reads	Library layout
**ULI-NChIP-seq (FGO)**		
H3K36me2_ctrl_rep1	89,210,770	53 paired-end
H3K36me2_ctrl_rep1_input	66,077,532	53 paired-end
H3K36me2_ctrl_rep2	122,427,330	53 paired-end
H3K36me2_ctrl_rep2_input	112,868,724	53 paired-end
H3K36me2_K36M_rep1	176,655,532	53 paired-end
H3K36me2_K36M_rep1_input	89,930,728	53 paired-end
H3K36me2_K36M_rep2	63,361,076	53 paired-end
H3K36me2_K36M_rep2_input	77,732,234	53 paired-end
H3K36me3_ctrl	95,691,462	53 paired-end
H3K36me3_ctrl_input	71,073,922	53 paired-end
H3K36me3_K36M	80,520,322	53 paired-end
H3K36me3_K36M_input	79,697,516	53 paired-end
**CUT&RUN (FGO)**		
H3K27me3_ctrl_rep1	93,945,052	53 paired-end
H3K27me3_ctrl_rep2	356,237,456	53 paired-end
H3K27me3_K36M_rep1	311,634,240	53 paired-end
H3K27me3_K36M_rep2	116,577,938	53 paired-end
**RNA-seq (FGO, 2C)**		
RNA_FGO_ctrl_rep1	95,414,674	53 paired-end
RNA_FGO_ctrl_rep2	105,769,228	53 paired-end
RNA_FGO_ctrl_rep3	97,972,924	53 paired-end
RNA_FGO_K36M_rep1	78,367,432	53 paired-end
RNA_FGO_K36M_rep2	110,243,364	53 paired-end
RNA_FGO_K36M_rep3	93,777,790	53 paired-end
RNA_2C_ctrl_rep1	35,759,344	53 paired-end
RNA_2C_ctrl_rep2	39,112,794	53 paired-end
RNA_2C_ctrl_rep3	34,484,476	53 paired-end
RNA_2C_ctrl_rep4	32,954,410	53 paired-end
RNA_2C_ctrl_rep5	29,292,670	53 paired-end
RNA_2C_ctrl_rep6	27,539,238	53 paired-end
RNA_2C_ctrl_rep7	30,680,086	53 paired-end
RNA_2C_ctrl_rep8	32,776,216	53 paired-end
RNA_2C_K36M_rep1	30,665,830	53 paired-end
RNA_2C_K36M_rep2	29,135,982	53 paired-end
RNA_2C_K36M_rep3	39,490,158	53 paired-end
RNA_2C_K36M_rep4	28,632,776	53 paired-end
RNA_2C_K36M_rep5	25,398,318	53 paired-end
RNA_2C_K36M_rep6	28,246,600	53 paired-end
RNA_2C_K36M_rep7	35,072,658	53 paired-end
RNA_2C_K36M_rep8	22,671,142	53 paired-end

Number of total reads and that of reads mapped to spike-in (SNAP-ChIP-Kmet Stat Panel)

	control	*K36M*	*K36M* /control
H3K36me2_rep1			
Total reads	88,151,570	173,903,282	
Spike-in reads	1,589	3,630	
Total/spike-in	55476.130	47907.240	**0.864**
H3K36me3_rep1			
Total reads	93,998,664	79,341,810	
Spike-in reads	5,736	4,445	
Total/spike-in	16387.494	17849.676	**1.089**

Number of reads mapped to spike-in with specific histone modifications (for validation of antibody specificity)

	ChIP	input	ChIP/input	ChIP/input (normalized to maximum value)
H3K36me2_control_rep1				
H3K36me1	24	37	0.649	**0.211**
H3K36me2	77	25	3.080	**1.000**
H3K36me3	12	52	0.231	**0.075**
H3K36me3_control_rep1				
H3K36me1	48	61	0.787	**0.068**
H3K36me2	63	66	0.955	**0.082**
H3K36me3	944	81	11.654	**1.000**

Supplementary Table 3. Summary of WGBS in FGO.

Sample	Total reads	Library layout	Mapping efficiency (%)	Average depth per strand	Conversion rate (%)	CG methylation (%)	non-CG methylation (%)
WGBS_ctrl_rep1	493,864,036	108 single-end	59.6	7.5	99.5	36.2	3.1
WGBS_ctrl_rep2	317,267,573	108 single-end	51.2	2.3	99.3	35.8	3.0
WGBS_K36M_rep1	496,433,476	108 single-end	59.4	7.6	99.4	30.8	2.7
WGBS_K36M_rep2	332,173,968	108 single-end	59.6	2.8	99.3	30.3	2.7
WGBS_setd2_KO	496,916,144	108 single-end	52.5	6.8	99.6	18.8	0.7
WGBS_DM_rep1	406,576,152	108 single-end	60.4	6.0	99.5	7.5	0.5
WGBS_DM_rep2	443,662,421	108 single-end	60.2	6.8	99.6	7.5	0.5

Supplementary Table 4. Oligonucleotide primers used in this study.

K36M_F	GCCTTGAACGTCGCTTGTCTCGCAG
K36M_R	CCACCAGGTATGCTTCGCTAGCCTC
Setd2_F	GAGCTCATTGTCAACACAAACAG
Setd2_R	TTCTGGGAATCATCCATGGT
Zp3-Cre_F	GCAGAACCTGAAGATGTTCGCGAT
Zp3-Cre_R	AGGTATCTCTGACCAGAGTCATCC
Gdf9-Cre_F	TCTGATGAAGTCAGGAAGAACC
Gdf9-Cre_R	GAGATGTCCTTCACTCTGATTC
