# Supplementary material for: Histone H3K36me2 and H3K36me3 form a chromatin platform essential for DNMT3A-dependent DNA methylation in mouse oocytes
Source: Nat Commun. 2022 Aug 3;13:4440. doi: 10.1038/s41467-022-32141-2 (PMC9349174; doi:10.1038/s41467-022-32141-2)
Supplement: Supplementary file 3 — Description of Additional Supplementary files [file 41467_2022_32141_MOESM3_ESM.pdf]

Histone H3K36me2 and H3K36me3 form a chromatin platform essential for DNMT3A-dependent DNA methylation in mouse oocytes

Seiichi Yano, Takashi Ishiuchi, Shusaku Abe, Satoshi H. Namekawa, Gang Huang, Yoshihiro Ogawa, Hiroyuki Sasaki

Description of Supplementary files

Supplementary Data 1. List of genes with H3K36me2 loss
Supplementary Data 2. List of genes with H3K36me2 enrichment
Supplementary Data 3. List of genes with H3K36me3 enrichment
Supplementary Data 4. List of differentially expressed genes
